# Learning from COVID-19: government leaders’ perspectives to improve emergency risk communication

**DOI:** 10.1186/s12992-023-00993-y

**Published:** 2023-11-16

**Authors:** Elena Savoia, Rachael Piltch-Loeb, Eva H. Stanton, Howard K. Koh

**Affiliations:** 1 Department of Biostatistics, Harvard T.H. Chan School of Public Health, 677 Huntington Avenue, Boston, MA 02115 USA; 2 Emergency Preparedness Research Evaluation and Practice (EPREP) Program, Division of Policy Translation and Leadership Development, Harvard T.H. Chan School of Public Health, 677 Huntington Avenue, Boston, MA 02115 USA; 3 Department of Health Policy and Management, Harvard T.H. Chan School of Public Health, 677 Huntington Avenue, Boston, MA 02115 USA

**Keywords:** Emergency risk communication, COVID-19, Government response, Principles

## Abstract

**Background:**

The coronavirus disease 2019 (COVID-19) pandemic highlighted the challenges of effective emergency risk communication (ERC) to protect public health, including the difficulty in tackling the spread of inaccurate information. This study aimed to understand those challenges and potential solutions by interviewing leading government spokespersons and their advisors from around the world with experience during large scale emergencies. Interviews were conducted with 27 individuals representing governments from 19 countries across five continents. Thematic analysis, using both a deductive and inductive approach, organized and identified salient themes and patterns that emerged from the interview data.

**Results:**

The thematic analysis of the interviews’ data led to the identification of 9 principles of communication: 1) Timeliness, 2) Transparency, 3) Coordination, 4) Accuracy and Consistency, 5) Accountability and Integrity, 6) Independence from politics, 7) Responsiveness, 8) Equity, 9) Trust and Empathy. We also developed 36 recommendations actionable by government agencies to enhance the practice of the 9 principles. Examples include the need for: proactive communication strategies, permanent communication task forces integrated into preparedness and response efforts, robust processes to enhance open discussion of controversial topics within government agencies, clarification of how various branches of government coordinate to oversee specific aspects of the overall communication, and development of relationships across public and private entities ahead of a crisis.

**Conclusions:**

Our findings suggest key practical recommendations for leaders of government agencies to enhance ERC capabilities going forward. Before a crisis, they must constantly review internal processes and integrate ERC functions into overall communication planning efforts. During a crisis, they must coordinate roles and responsibilities across branches of governments, strive to communicate to a range of populations to uphold equity, maintain transparency by avoiding information voids on controversial issues and build trust by building relationships with a variety of community leaders. After a crisis, government agencies should continue the practice of social listening to hear more about the public’s informational needs, strengthen civic participation processes, and understand how an always evolving information environment can best be leveraged during future crises.

## Background

During the COVID-19 pandemic, government agencies around the world mobilized unprecedented resources to deliver effective emergency risk communication (ERC) to the public [1]. Yet, despite substantial government efforts, false information regarding virtually every aspect of the overall response, such as medical treatment options and vaccine safety, spread fear and skepticism among some segments of the population. This reaction, while expected due to the multiple factors that shape how people perceive and act upon the information they receive during crisis situations, was particularly heightened through the pandemic. Malecki et al. note that “Public perception of risk in a pandemic is shaped by not only the true nature of the hazard but includes various contextual outrage factors” [2]. Among factors influencing risk perception are values stemming from the social and cultural context in which people live, the immediacy and unfamiliarity of the threat, the uncertainty of the situation and its related risk for the individual, self-efficacy, and control over the risk of exposure, as well as levels of trust in government and non-governmental institutions and in the sources of information. All these factors mediate the way people perceive risk, understand communication messages, and decide whether to comply with recommended behaviors issued by government agencies [3].

### Principles of communication

In the past two decades, public health emergencies– such as the anthrax attacks, SARS, Ebola, Zika, and more recently, COVID-19 – have prompted national and international agencies to offer and update guiding principles for government officials in charge of public communication efforts. Table 1 summarizes four guiding documents developed over the years by the US Centers for Disease Control and Prevention (CDC), the Organization for Economic Cooperation and Development (OECD), the World Health Organization (WHO) and the European Centre for Disease Control and Prevention (ECDC).

**Table 1:** Guiding documents

Agency	Document Title	Date	Principles Listed
Centers for Disease Control and Prevention (CDC)	Crisis and Emergency Risk Communication (CERC) Manual [4]	2002 (last update in 2019)	• Be First • Be Right • Be Credible • Express Empathy • Promote Action • Show respect
Organization for Economic Cooperation and Development (OECD)	Principles of Good Practice for Public Communication Response to Mis- and Disinformation [1].	2022	• Institutionalization • Public interest driven • Future proofing and professionalization Transparency • Timeliness • Prevention • Evidence-based • Inclusiveness • Whole of society collaboration
World Health Organization (WHO)	Communicating risk in public health emergencies. A WHO guideline for emergency risk communication (ERC) policy and practice [5].	2018	• Building trust and engaging with affected populations • Integrating emergency risk communication into health response systems • Emergency risk communication practice
European Centre for Disease Prevention and Control (ECDC)	Public health emergency preparedness – Core competencies for EU Member States [6].	2017	• Communicate risk in a timely and transparent manner • Foster and maintain trust with the media and the public • Communicate risk in a clear, consistent, and empathetic manner • Identify and address communication inequalities

To our knowledge, the first set of ERC principles was published in 2002 by the CDC in their Crisis and Emergency Risk Communication (CERC) manual [4]. From 2014 to 2019, the manual was updated several times to guide public health professionals in public communication efforts. In 2021, the OECD opened a public consultation process on principles of good practice in public communication to promote capacity-building activities in managing mis-disinformation, adopting the definition of information disorder developed by Wardle and Derakshan [7], reported in Table 2.

**Table 2:** Information disorder [7]

Misinformation	“when false information is shared, but no harm is meant”. This consists typically of rumour or misleading content shared unknowingly by individuals.
Disinformation	“when false information is knowingly shared to cause harm”. Disinformation can often be traced back to actors with malicious motives and can be part of concerted large-scale campaigns.
Malinformation	“when genuine information is shared to cause harm, often by moving what was designed to stay private into the public sphere”

The OECD effort was driven by pre-pandemic data documenting that governments often fail to effectively communicate with and engage citizens, reflecting a lack of preparedness capabilities in public communication. In fact, OECD data showed that only 38% of Central Governments and 21% of Ministries of Health had a guiding document in place to govern the response to mis- and disinformation at the outset of the pandemic [8].

While the CDC and OECD documents are the only ones to explicitly cite principles of communication, other international organizations have developed recommendations for ERC. In 2017, WHO released a guideline for ERC policy and practice with recommendations highlighting the importance of building trust, establishing processes to engage the public during a crisis, and integrating ERC functions in the overall government response [5]. The WHO guidelines emphasized the need to update governments’ communication efforts for the twenty-first century in light of the “near-universal penetration of mobile telephones, the widespread use and increasingly powerful influence of digital media, and based on populations’ behaviors on how they search for information and the messengers they trust” [5].

Finally, the ECDC developed a preparedness competency model to specify response capabilities, preparedness capacities, and competencies necessary to achieve ERC as a critical part of preparedness efforts [6]. During COVID-19, the ECDC model was updated to include the capability of infodemic management [9].

Future responsibility for effective ERC will continue to fall on leading government officials from around the world, such as cabinet office members, ministers of health, chief medical officers, and prominent subnational figures. However, to date, few studies have documented where current ERC principles should be updated to best address future threats in the most practical ways possible [10–12].

Two practice-based research questions drove this study: 1) What communication challenges for government leaders worldwide complicated the COVID-19 response and ability to adhere to ERC principles of communication? and 2) What practical recommendations can improve ERC planning efforts for future crises?

## Methods

Our methodology consisted of semi-structured interviews conducted via Zoom between August and November 2022, as well as an in-person meeting conducted in September 2023.

### Interviews

We interviewed 27 current (as of October 2022) and former leading government officials with experience as the spokesperson –or the direct advisor to the spokesperson— during public health emergencies, focusing on, but not limited to, the COVID-19 pandemic. Interviewees were selected based on snowball sampling [13] with the objective to purposefully identifying government officials with relevant experience in communicating with the public during a public health emergency. Initial participants, identified from a list of individuals known to the research team from prior ERC work, then recommended peers in the field for interview. Sampling concluded when two states were achieved: 1) saturation of content, defined as reaching a state of repetition across topical areas of inquiry, and 2) diversity in the geographic locations of the countries and their size represented in the sample.

Interviewees represented governments from 19 countries, with working experience at the national and sub-national level across the five continents (Table 3). The interviewees represented governments of different sizes, from the smallest nation in our sample, like San Marino (with approximately 33,000 residents), to the United States, the largest in our sample, with a population of over 300 million.

**Table 3:** Number of interviewees by country (19 countries) and organizations they worked for in their leadership role

Countries 26 interviewees were current or former government officials + 1 interviewee from WHO	Organizations and leadership roles (*N* = 1 unless otherwise specified)
Armenia (1) Brazil (1) Croatia (1) France (1) Germany (1) Indonesia (1) Israel (2) Italy (2) Japan (1) North Macedonia (1) Malta (1) Nigeria (1) Norway (1) Qatar (1) San Marino (1) Serbia (1) Sweden (1) United Kingdom (2) United States (5)	Cabinet Office Central Government, National Department of Network and Partnerships Country Mission to the European Union Health Promotion and Disease Prevention Country Directorate Large Metropolitan City COVID Task Force Large Metropolitan City, Emergency Medical Services Large Metropolitan City, Public Health Commission National Agency for Community Safety and Preparedness National COVID Taskforce National Government Crisis Information Unit National Institute from Social Security National Medicine and Medical Devices Agency National Ministry of Health (*N* = 9 interviewees) National Ministry of Communication and Informatics Prime Minister’s Office Sub-national Institute of Public Health (*N* = 2 interviewees) U.S. Department of Homeland Security/Federal Emergency Management Agency U.S. Department of Health and Human Services • Administration for Strategic Preparedness and Response • Centers for Disease Control and Prevention (N = 2 interviewees) • Indian Health Service • Office of the Assistant Secretary for Health World Bank World Health Organization

All individuals included in this sample were recruited because of their technical role in health communication at the government level. Examples of leadership roles represented by the interviewees at the national level include Executive Director for Communication in the Prime Minister's Cabinet Office, Chief Medical Officer, Head of Communications, and Minister of Health; the specific role of any specific individual was not listed for confidentiality purposes. Examples of leadership roles assumed by the interviewees at the sub-national level include Commissioner of Health at the state and county level, Assistant Director of the public health institute of a large metropolitan city, and Emergency Medical Services Chief. A list of organizations interviewees is provided in Table 3; the link between role and country has been omitted to ensure confidentiality.

### Interview guide and methods

The interview guide was developed based on the principles of communication created by the CDC and OECD (Table 1). The research team independently took notes on the specific principles mentioned in each document and met to select common principles to include in the interview guide. Based on the purpose of this study, the team prioritized and selected principles that reflected ethical standards for good practice in communication that are applicable and relevant at the spokesperson level; principles and recommendations related to system-level resources, organizational structures, or the institutionalization of ERC functions at the government level were excluded from the interview questions. This process reflected the goal to develop a concise list of principles that could be reasonably addressed during a 60–90 min interview timeframe. Principles included in the interview guide were timeliness, transparency, accuracy, communication of uncertainty, consistency, integrity, accountability, listening and civic participation, ethics, and equity. The interviewer, started with questions on the interviewee’s role and the organizational structure of ERC efforts in their government, asked interviewees to speak to the relevance of the list of selected principles generated by the research team as they pertained to the management of information disorder (defined in Table 2) during COVID-19 and other emergencies. They were also asked to recommend any principles possibly missing from the list and provide examples of ERC practices to better address information disorder while adhering to communication principles.

### Analysis

All interviews were transcribed verbatim in their original language (English, Portuguese, Italian, French, Serbo-Croatian, and Macedonian), translated into English, and coded from the English version. For the interviews conducted in a language other than English, the research team member - who spoke the language of the interviewee - reviewed the recording of the interview and the results of the coding to ensure the fidelity of the translation and its interpretation. Thematic analysis, following standard qualitative analysis guidelines [14] organized and identified salient themes and patterns that emerged from the interviews’ data. Three research team members independently read and coded the interviews as part of an iterative approach to establish a framework of response patterns. Specifically, team members first familiarized themselves with the raw data and generated initial codes in relation to the list of principles included in the interview guide (deductive approach), and then generated new codes based on the interviewees’ responses (inductive approach). Subsequently, the research team worked collectively to group the codes into descriptive themes that captured the core meaning of the included content while also selecting specific interviewees’ quotes to capture each theme. Consensus on the final list of themes (principles) and respective quotes was achieved through multiple peer debriefing meetings.

### In-person meeting

Finally, in an in-person international meeting, the team elaborated a list of practice recommendations within each theme (principle) derived from interview results of the interviews and presented to a group of eight government officials-- 5 were interviewees from which the recommendations were elaborated, and three were in leadership roles working for the United Nations Global Fund Programme, WHO, Ministry of Health in Portugal, and Privy Council Office of the Canadian Government; hence, two more countries were added to our sample: Portugal and Canada. These eight leaders rated the recommendations elaborated by the team by level of importance (ranging from 1 [very low importance] to 10 [very high importance]) and provided comments when needed. The average score and standard deviation for each recommendation were calculated to determine consensus on their level of importance.

## Results

Thematic analysis of the interviews’ data led to the identification of 9 principles of communication: 1) Timeliness, 2) Transparency, 3) Coordination, 4) Accuracy and Consistency, 5) Accountability and Integrity, 6) Independence from politics, 7) Responsiveness, 8) Equity, 9) Trust and Empathy.

In the following sections, after briefly describing the meaning of each principle as explained in the international guiding documents already noted, we cite the challenges to these principles in practice as reflected in specific quotes from the interviewees. For each quote included in the results, we provide the corresponding recommendation, delineated in brackets as *[recommendation x]*, that the research team elaborated from the interviews’ data. The 36 recommendations elaborated by the team are listed in Table 4. For each recommendation, we provide the average rating and standard deviation, describing its level of importance as determined by a group of government officials participating in an in-person meeting.

**Table 4:** Recommendations to improve ERC practice

Recommendations to improve the practice of ERC principles - average rating (standard deviation)	
**1. TIMELINESS**	
1a - Acknowledge the level of scientific uncertainty supporting specific information released to the public and the likelihood that it will change over time.	9 (2.5)
1b - Improve the speed of the decision-making process and related communication activities in public health.	8.2 (0.9)
1c - Improve the speed of the government-level clearance process for issuing messages to the public.	8.4 (1.8)
1d - Communicate at regular intervals.	8.7 (1.8)
1e - Develop government communication strategies focused on leading the narrative.	7.6 (2.4)
**2. TRANSPARENCY**	
2a - Embrace transparency in communicating what is known and still unknown despite the potential economic, social, and political consequences.	8.4 (1)
2b - Communicate the decision-making process behind specific preventive measures and the interpretation of the science supporting the measures.	7.5 (1.2)
2c - Inform the public of how social listening activities are being conducted.	6.6 (1.1)
2d - Develop internal government processes so topics are discussed openly within government staff (intra-agency transparency).	8.7 (1.5)
**3. COORDINATION**	
3a - Create permanent task forces that integrate expertise in public communication.	9.2 (0.7)
3b - Create a centralized internet presence (i.e., dashboards) with plans to keep the information up to date and accurate.	8.6 (1.1)
3c - Prioritize the role of the government agency that is the closest - geographically and culturally- to the affected population when releasing the message to the public.	6.2 (1.5)
3d - Build relationships and coordination across different branches of government in charge of the release of information to the public as well as with government agencies in neighboring countries.	9 (1.2)
**4. ACCURACY AND CONSISTENCY**	
4a - Develop processes to update web pages dedicated to FAQs.	8.6 (1.9)
4b - Customize FAQs to the needs of different types of audiences.	8.6 (1.3)
4c - Translate scientific information into plain language prior to delivering it to the political appointees.	9.2 (1.4)
4d - Increase awareness in spokespersons and political figures of the importance of following the preventive measures they recommend in their personal life.	8.5 (2)
**5. ACCOUNTABILITY AND INTEGRITY**	
5a - Communicate the decision-making process behind the recommendations issued to the public.	7.7 (1.1)
5b - Acknowledge mistakes and delays as they occur.	9 (1.2)
5c - Engage with media outlets across the political spectrum.	8.5 (0.9)
5d - Discern a priori the responsibility of different branches of government when communicating to the public.	6.8 (1.2)
5e - Develop evaluation processes to determine the effectiveness and consequences (positive and negative) of communication practices.	8.6 (1.7)
5f - Address population health and communication inequities to prepare for future crises.	9.5 (2.4)
**6. INDEPENDENCE FROM POLITICS**	
6a - Separate the scientific communication from the political communication regarding implementation of specific policies.	8.6 (0.7)
6b - Be on guard for the risk of politicization of policies in particular when the government lacks the ability to enforce them.	8.5 (0.9)
6c - Avoid the use of entertainment venues (i.e., TV talk-shows) and similar platforms to announce new policies and the forthcoming policy changes.	6.6 (1.2)
**7. RESPONSIVENESS**	
7a - Acknowledge the priorities of the population.	8.6 (1.2)
7b - Develop networks of community leaders and professional figures that can inform the government on the population’s informational needs and support government communication efforts.	8.6 (1.1)
7c - Partner with private companies (i.e., social media companies) to enhance communication efforts and outreach to specific audiences.	7.7 (0.9)
**8. EQUITY**	
8a - Develop communication strategies that account for diversity in linguistic background, health and digital literacy, internet access and culture.	9.5 (2.1)
8b - Build partnerships with local leaders who may be able to reach specific audiences.	9.4 (1.7)
8c - Develop education campaigns to enhance digital literacy and the public’s ability to discern misinformation.	9.2 (2.7)
8d - Engage with communities before there is a crisis to understand their pre-existing informational needs and priorities.	8.6 (3.3)
**9. TRUST AND EMPATHY**	
9a - Develop strategies to establish trust at different levels ahead of a crisis (i.e., trust in government, between citizens, among different levels of government, between the private and public sector).	9.9 (1.2)
9b - Create opportunities to build trusted relationships between different branches of government and entities before and during a crisis (i.e., in person- visits to affected areas).	9 (1.1)
9c - Validate people’s feelings and fears when recommending practices, they are concerned about.	9 (0.3)

### Timeliness

*“Crises are time sensitive…for members of the public, the first source of information often becomes the best source*.” CDC CERC [4].

Fourteen interviewees, in citing timeliness as a key communication principle, noted the major tension between timeliness and accuracy during a crisis with regular uncertainty about always evolving scientific evidence complicating the information environment. As a United States interviewee recognized “... *even right now [two years after the start of the crisis], we’re still not doing a good job in telling people: this is what we know now, and that it’s going to change again tomorrow”. [recommendation 1a]* The same interviewee noted that since the culture surrounding the public health decision- making process typically involved the expectation of bringing multiple stakeholders together in often time-consuming processes, such expectations about consensus building frequently delayed and/or complicated government communication. *[recommendation 1b].*

An interviewee from Serbia noted the contrast between the lengthy internal clearance process for government release of information to the public versus the rapidity of misinformation spread by social media. This interviewee noted that after inaccuracies are detected, “*it takes like a day or two to come up with a very scientific sound response… which subsequently needs to undergo a lengthy clearance”*. *[recommendation 1c].* An interviewee from Croatia emphasized the importance of having regular meetings among agencies involved in government communication to synchronize efforts internally: “*we had a strategy of “holding” regular meetings of the Crisis Unit so that everyone had the same information “at the same time” to deliver to the public”. [recommendation 1d]* One interviewee from Brazil also emphasized the importance of synchronicity externally with the public by communicating to the public at a regular predetermined frequency. *[recommendation 1d].*

Finally, an interviewee from Israel noted that government should be proactive in *“leading the narrative* “rather than routinely reactive to the misinformation disseminated by various types of media” *[recommendation 1e].*

### Transparency

*“Governments should strive to… comprehensively disclose information, decisions, processes, and data within the limitations of relevant legislation and regulations. Transparency, including about assumptions and uncertainty, can reduce the scope for rumors and falsehoods to take root,”* OECD [1].

Twelve interviewees emphasized the importance of transparent communication even in the face of incomplete information and high potential for negative public reactions. An interviewee from Japan underscored that the negative dimensions of public health emergencies and its attendant adverse economic and social impacts complicate people’s ability to process the information received *“... if the price is too high, they will not be able to understand it yet”*. *[recommendation 2a].*

All interviewees agreed that the best transparency strategy was to acknowledge what is known and still unknown, and explain the rationale behind the decision-making process related to recommended preventive measures [*recommendation 2a and 2b].*

An interviewee from Nigeria discussed the importance of being transparent on the mechanisms used for social listening, i.e., gathering data at the population level to understand their informational needs and concerns. This interviewee noted that governments must *“…do [social listening] in a way that is governed properly, that is transparent, that is inclusive, that people know what information is collected and what it is being used as identifiers*.” *[recommendation 2c].*

An interviewee from the United States stated the importance of internal transparency within government agencies since if controversial topics are not openly discussed “*it looks like we’re trying to avoid the question. We are (only) answering the question that we hope they’d (the public would) ask*.” *[recommendation 2d].*

Interviewees also noted that despite the potential negative consequences of transparent communication at the economic, social, and political levels, the positive impacts outweighed the negative ones over the long term. As one interviewee from Nigeria remarked, *“…. no matter how you spin it [the information you are withholding from the public] or you de-emphasize it, or you ignore it, it will all come back to haunt you*.” *[recommendation 2a].*

### Coordination

*“Crisis coordination [is] synchronized information sharing between response organizations… Emergency communications and response efforts depend on… careful coordination,”* CDC [4].

Fourteen interviewees, in citing coordination as a critical communication principle, emphasized that crisis task forces should routinely include communication experts regarding how best to convey public policy decisions. An interviewee from Italy suggested that such integration should start in the pre-pandemic phase and be made permanent before any crisis begins. *[recommendation 3a].*

Coordination can occur at multiple levels. To improve coordination between national and local governments during a crisis, interviewees from Italy and Germany recommended creating, during a crisis, a centralized internet presence (i.e., dashboards) for the release of daily situation reports and committing adequate resources to keep web-based information up to date and accurate (i.e., data quality control processes) *[recommendation 3b]*. Regarding coordination between national and local level agencies, an interviewee from a local agency in Croatia argued that the government agency closest to the population being impacted should be the first to release information to them. This interviewee shared an example whereby COVID-19 results on swab samples, sent by a local agency to a national laboratory that determined them positive for COVID-19, were publicly released by the latter organization instead of the former. As the interviewee noted “... *we may have left a feeling of insecurity in the population, that is, that we do not have accurate or do not know the information.*” *[recommendation 3c].* Further, an interviewee from Brazil highlighted the importance of government-wide cohesion, stressing that all government branches need to be informed about: *“who is going to communicate what (i.e., surveillance data, logistical information, overall situation), what role they have (sometimes technical and sometimes political role) and what level of responsibility… A synchronized response.” [recommendation 3d].*

Coordination is also critical between countries, especially of neighboring nations. An interviewee from Armenia noted, *“...if we worked better with our neighbors…it would have helped because we would have been able to share information together.” [recommendation 3d]*. An interviewee from Norway noted the importance of both formal and informal information-sharing mechanisms based on long-term pre-existing trusted relationships: *“You need to know their face, and you need to trust. And then you can alarm each other when you know you are short on time, you can’t wait for a paper or go through the system – you just have to tell them*.” *[recommendation 3d].*

### Accuracy and consistency

*“... the use of consistent messaging via different information sources in an emergency increases the likelihood that messages will be believed and acted upon… absent or contradictory and inconsistent information from the authorities leads to uncertainty.”* WHO [5].

Of the nine interviewees that cited the importance of consistent communications, one from Malta noted that communication inconsistencies are *“quickly picked up and amplified by the media”*, confuse the public and damage the credibility of government spokespersons. While interviewees overall noted that messaging inconsistency almost inevitably regularly arise due to the natural evolution of the scientific process, different interpretations of scientific findings, and the need for policies to be adapted to the local needs, they cited instances where inconsistencies could have been easily prevented. For example, an interviewee from Italy stated that government agencies were very efficient in rapidly creating web pages to post answers to COVID-19 frequently asked questions (FAQs), but then lacked processes to generate regular information. An interviewee working at the international level noted: *“...if we don’t take down outdated information, that fuels misinformation because people that really want to misuse information that’s on the internet can literally say that the health authority is inconsistent in communicating.” [recommendation 4a]*. The same interviewee pointed to the lack of customization of FAQs to meet people’s informational needs, noting: *“... a conspiracy theorist and a medical doctor looking for factual information are both expected to get an answer from the same FAQ”. [recommendation 4b].*

An interviewee from Japan also noted that inconsistency can arise when politicians not well briefed a priori by their scientific experts incorrectly translate and then communicate technical scientific content into plain language. *[recommendation 4c].*

Finally, an interviewee from Croatia emphasized the importance of consistency between words and personal behaviors, arguing that government officials should show consistency between what they recommend professionally and do personally (i.e., referring to COVID-19 the interviewer talked about wearing a mask and refraining from social gatherings in their personal life). *[recommendation 4d].*

### Accountability

*“More continuous and committed efforts to listen to and understand public sentiment… can contribute to greater accountability and responsiveness*.” OECD [1].

Seven interviewees emphasized that a crisis demands accountability practices beyond those determined by law. The interviewees highlighted the need to provide the public with explanations about the decision-making process behind newly released policies especially when they are based on limited or contradictory scientific evidence. *[recommendation 5a].*

The need for apologies and corrections when mistakes occur was acknowledged by interviewees from San Marino and North Macedonia. *[recommendation 5b]* An interviewee from Serbia reported that accepting invitations to be interviewed by journalists with politically opposing views is an opportunity to address criticism and practice social accountability. *[recommendation 5c].*

Agencies regularly face challenges when discussing issues that are outside of their mission and area of technical knowledge. An interviewee from the United States emphasized the need to discern what falls outside the purview of one’s public health institution and determine ahead of time “... *what is within your wheelhouse, what is your role as an institution (government institution) versus the role of another institution”. [recommendation 5d].*

An interviewee working at the international level spoke about the importance of implementing evidence-based communication practices to ensure government accountability. *[recommendation 5e]* On this matter, a government official from the United Kingdom highlighted the usefulness of establishing rapid evaluation mechanisms to hold governments accountable for the information they decide to release and funds they invest in related public outreach campaigns. *[recommendation 5e].*

Finally, an interviewee from the United States spoke about the need for government agencies to assume accountability not only in response to current emergencies but also “*... thinking ahead of events*” for the future, with a special obligation to reduce social inequities that may impact future crises. *[recommendation 5f].*

### Independence from politics

*“Public communication should strive to be independent from politicization in implementing interventions to counteract mis- and dis-information. Public communication should be separate and distinct from partisan and electoral communications,”* OECD [1].

Of thirteen interviewees that cited such independence as a key principle, one from the United States remarked that when a health decision becomes politicized, the health authority quickly loses control over the information being conveyed, saying, “*.... at that point, you just have limited influence*.” Therefore, creating a non-partisan context is critical. For example, during COVID-19, specific segments of the population refused vaccination in part because politicians with political views opposed to theirs advocated for it, as remarked by an interviewee in the UK.. A government official from Italy spoke of the need of separating the scientific spokespersons from the political appointee during public speech. Having political appointees and administrators explaining the operational aspects of specific policies (i.e., what services are open and closed during a lockdown), leaving all related scientific explanations around such policies to the public health technical experts. *[recommendation 6a].*

An interviewee from the United Kingdom argued that vaccination mandates challenged communication when enforcement was viewed as unachievable. Similarly, an interviewee from Germany reported that the contrast between the broad scope of a policy recommendation and the government’s limited ability to execute it frequently fueled attention to politics, not health. *[recommendation 6b].*

Furthermore, an interviewee from Germany expressed concerns that some political decisions were announced during TV talk shows, creating a media effect further politicizing health matter “*leadership has become more of a performance artist that has undercut the trust in government institutions …. so, it’s very difficult for anyone who represents a government to overcome that sort of bias*.” *[recommendation 6c].*

### Responsiveness

*“Effective risk communication allows… authorities and experts to listen to and address people’s concerns and needs so that the advice they provide is relevant, trusted and acceptable.”* WHO [5].

Of the 20 interviewees who highlighted responsiveness, one from the United States suggested that *“you have to engage with the community, discern what their concern is and then bring them into the picture to work on the problem that they see.” [recommendation 7a].* Another interviewee from Nigeria noted that listening can enable government officials to understand that focusing on a single issue (i.e., vaccination) may not be reflective of community priorities, saying *“...when the government emphasizes a singular issue, it can inadvertently undermine itself*” *[recommendation 7a]* and that *“... that is how those who derail people actually get this misinformation out because they do that (listening) perhaps even better than those of us who are trying to do the right thing*.”

When discussing listening tools, interviewees from France, Italy, Serbia, and Qatar described helplines as useful for monitoring the population’s informational needs. An interviewee from Croatia also cited the usefulness of national polls to identify public concerns, behaviors, and misinformation endorsement. *[recommendation 7a].*

Interviewees also discussed the importance of building networks of community leaders and professional figures (i.e., religious, business leaders, academic institutions) who can share with government officials the concerns of the community and support efforts to the disseminate messages to specific audiences. An interviewee from Serbia noted: *“... people will simply not believe me, but they will believe our religious leader, they will believe our local politician or some other authority, maybe at the neighborhood level”. [recommendation 7b].* An interviewee from Nigeria emphasized that training of healthcare workers in communication helps reach diverse audiences “*People are … listening to the health worker that goes to their neighborhood and brings the vaccine. And if that health worker is not well prepared to answer their questions, that may be where they make that decision (of not getting vaccinated).” [recommendation 7b]* Furthermore, an interviewee from the U.S. discussed the need for public-private partnerships, referring to social media companies that have the tools to disseminate the information, monitor informational needs, and identify misleading informational narratives. *[recommendation 7c].*

### Equity

*“The benefit of specific, culturally, and contextually appropriate actions that people can take themselves during an emergency was emphasized… Equity– providing realistic actions to vulnerable groups– was an important element…”* WHO [5].

Of the 9 interviewees that cited equity as a key communication principle, one at the international level noted, *“Access to health information is a human right.”* Developing an equitable messaging strategy must account for variations in the linguistic background, health and digital literacy, internet access, and culture of the population. As an interviewee from Malta said, *“There were always the foreigners with the language problems, you know, who don’t hear our news, who don’t see the television, the radio, or read the newspapers and I used to try to use NGOs to help me”. [recommendation 8a and 8b].* An interviewee from Indonesia noted how such a grassroots approach aided digital literacy educational campaigns to recognize misinformation and reduce its spread. *[recommendation 8c].*

Efforts to increase access to information must start prior to an emergency. One interviewee working at the international level argued, *“It has to be a huge systemic effort through all health programs during peacetime to make sure that they get the services they need, and they get connected to the health system better. So, when the emergency hits, you have a shot.” [recommendation 8d].*

### Trust and empathy

*“One of the most important factors in effective communication is how much your audience trusts you and your organization. Establish trust through empathy and openness.”* CDC CERC [4].

Of the twelve interviewees citing trust as a critical communication principle, one from Croatia noted that the multiyear length of the pandemic, and related negative economic and social impacts, challenged the government’s ability to maintain public trust. Interviewees emphasized that trust needs to be built over time but trust, during this long pandemic was inevitably eroded. As an interviewee from Sweden described, *“I think it’s really important for a system as a whole to gain trust beforehand, because during the crisis you have to start paying... It’s like a bank account. And the minute the crisis hits, it’s like the central banks, they are now distributing money…*” *[recommendation 9a].*

Interviewees referred to multiple sites for trust---e.g., in government, in fellow citizens, in pharmaceutical products (i.e., vaccine), in the healthcare system, in politics, in scientists, and in science—as well as between national and sub-national government agencies, government, and media, and between the public and the private sector. An interviewee from the United States affirmed that trust building accompanies relationship building. He said that during a crisis, government officials should travel to the affected areas to talk in person to those leading the response and to the citizens affected: “*without the (in-person) relationship, it’s really hard to have that trust”. [recommendation 9b]* An interviewee from Sweden also noted the importance of building trust between fellow citizens *“We were constantly telling people that you have to behave a certain way, but we [the government] had to make them trust that we would do our share”,* and continued by saying *“... you have to start building trust in institutions before you come to the stage where you actually can avoid restrictions … I would argue that restrictions in themselves are actually diminishing the trust in institutions and between people. Because what society says using restrictions is that we cannot trust you”. [recommendation 9b]* Another interviewee from the United States highlighted the importance of validating people’s feelings, stating, *“... if you’re able to absorb some of their anger, validate some of it, then you can begin to talk about developing a trusted relationship so that your messaging gets through*.” *[recommendation 9c].*

## Discussion

During the past 20 years, major international agencies have set forth– and regularly updated --principles and guiding documents related to ERC practices for government leaders. This analysis, to our knowledge, is the first to specifically focus on lessons learned from government-level leaders applying ERC principles to the recent pandemic (and previous crises) to improve efforts in future emergencies. All study interviewees served in leadership positions at the national, local government, and/or international level with current (or former) major communication responsibilities during multiple public health emergencies (from the 2001 Anthrax attacks to COVID-19). Despite their differing positions, our results found common themes and practical insights that our research team elaborated into 36 recommendations (see Table 3) that can be used to develop or revisit communication plans, and strategies in preparation for future crises. We believe these study results, stemming from top government officials around the world, can add specificity that not only updates the existing large body of literature on ERC but also adds practicality in translating otherwise abstract principles of communication in advance of future crises.

Government leaders all indicated that efforts to improve communication capabilities must begin now, before the next crisis arrives. Government agencies must proactively and permanently integrate ERC plans and functions into operational response frameworks. Demonstrating ERC expertise, coordination across agencies and communication capabilities in daily activities must be the government norm, rather than representing an “add on” activity after the emergency response has begun. This must involve building a stable communication workforce over time, regardless of political administration, that can facilitate continuity of operations in communication capacity through successive public health emergencies. The increasing frequency of public health emergencies and the complexity of overlapping crises across the environmental, geo-political, and socioeconomic spectrum demands this commitment [15].

If such investments are made during routine activities, then during a crisis, government leaders would find themselves in a better position to implement communication roles and responsibilities, not only within their agency but also across different government organization. Such interagency processes should strive to enhance communication capabilities to reach a range of populations to uphold equity, maintain transparency and uphold trust. In addition, after a crisis, government agencies should continue the practice of social listening to hear more about the public’s informational needs, strengthen civic participation processes, and understand how the information environment is evolving and how it can be leveraged during future crises.

Regardless of the country and context, we found that conveying the principles of equity, trustworthiness, and transparency was challenging. Addressing these principles all throughout the disaster management cycle--before, during and after a crisis-- must meet the specific informational needs of all segments of the population, focusing on those most vulnerable to health risks, culturally and linguistically diverse communities and those most susceptible to misinformation [16, 17]. Most interviewees noted meeting these principles requires the expansion of communication strategies to better recognize the diversity of audiences, enhancement of the population’s digital and health literacy, creation of opportunities for public engagement, and partnership-building with a wide range of community leaders and organizations. Such longer-term efforts must begin before an emergency, so that relationships with leaders and trusted messengers can be effectively leveraged when an emergency unfolds.

Similarly, interviewees emphasized that trust represents an essential precondition for any population to embrace behavioral change, especially when government recommendations are based on incomplete or rapidly evolving science. Transparency is not only a predictor of, but also an avenue for, effective communication leading to trust in government and institutions. Related research suggests that “governments can build and sustain citizen trust by focusing on four areas: humanity, transparency, capability, and reliability” [18]. Moreover, a recent report from the National Academies of Sciences Engineering and Medicine (NASEM) notes that improving trust requires emphasis on consistency in the messaging, community engagement, a strong public health infrastructure and skilled workforce to support communication practices [19]. ERC leaders must recognize that the public may be more likely to listen to messengers who have previously built credibility and trustworthiness by being present, recognizable, and reliable in serving their communities in the past. A recent report published by OECD highlights how trust in institutions may be driven by cultural, socio-economic, and political drivers and the government’s capacity to address global and inter-generational issues [20]. Interviewees stressed the need for leveraging the resources of social media platforms into government agency efforts by building stronger public-private partnerships, creating a better-trained public health workforce on the evolving technology, and assuring transparent processes on how citizens’ social media data are being used.

After a crisis, interviewees noted the importance of government leaders conducting civic participation processes to empower citizens in bringing their society back to “normality” based on listening, dialogue, and open discussion. Prior research notes that the public judges government performance based on how it addresses the key issues most important to them [21]. Therefore, understanding the public’s priorities after an event can contribute to better communication in future crises. More specifically, it is important to proactively engage with the public to understand their informational needs in areas that might be the object of communication challenges in the future, such as the emergence of new technologies that could be deployed during a future emergency and require wider educational efforts at the population level.

### Study limitations and strengths

Our study includes limitations related to our methods and interpretation of the results. First, we only focused on the principles of communication and guiding documents elaborated by four main organizations: CDC, ECDC, WHO, and OECD, a limitation of which is the absence of a clear definition of what constitutes a principle. The initial selection of principles to be included in the interview guide was driven by the researchers’ interest rather than a structured assessment of the documents. The list of the 36 recommendations is the result of an effort to translate the results of the interviewees’ data into a practical tool by the research team. The recommendations are not a validated list elaborated by the interviewees. Future research should focus on gathering consensus and further elaborating the recommendations derived from this study. Finally, our sample was driven by a snowball methodology which can generate biased results due to shared experiences among participants. The results, from a diverse sample involving over several dozen countries, need to be confirmed in a larger sample and at this time may not necessarily be extrapolated to other contexts. Despite its limitations, this study presents three unique strengths, the results are based on the experience of individuals in leadership positions that have gained experience throughout the course of several public health emergencies, the analysis is based on data gathered right after the pandemic leveraging lessons learned from an event affecting the globe, and most importantly the results have been translated into recommendations for practice.

## Conclusions

This study highlights the importance of moving from the abstract principles of communication to practical recommendations for government leaders engaged in communication efforts during public health crises. This study provides 36 recommendations for practice elaborated from the results of a series of interviews with 27 government officials across 19 countries. Future research efforts should aim to measure the impact of such recommendations on public health emergencies response efforts.

## Data Availability

Due to the qualitative nature of the data, the IRB procedures, and the ease with which respondents might be identified based on the content of the transcripts, we are unable to make the interview transcripts publicly available.

## Abbreviations

CDC: Centers for Disease Prevention and Control
CERC: Crisis and Emergency Risk Communication
ECDC: European Centre for Disease Prevention and Control
ERC: Emergency Risk Communication
FAQ/s: Frequently asked question/s
NGO: Non-governmental organization
OECD: Organization for Economic Cooperation and Development
WHO: World Health Organization
